# Assessing resident participation and its effect on postoperative complications in intertrochanteric and femoral neck fractures: insights on orthopaedic residency training in the USA—A NSQIP retrospective study

**DOI:** 10.1007/s00590-025-04554-4

**Published:** 2025-10-10

**Authors:** Majd Mzeihem, Joseph A. Karam, Jibreel Hussain, Jason Koh, Farid Amirouche

**Affiliations:** 1 University of Illinois at Chicago, Chicago, USA; 2 NorthShore University HealthSystem, Evanston, USA

**Keywords:** Hip fracture, Intertrochanteric fracture, Femoral neck fracture, Resident training, NSQIP database, Surgical education

## Abstract

**Introduction:**

The global incidence of hip fractures continues to rise with an aging population. In the USA, orthopedic residency programs prioritize surgical mentorship to prepare trainees for independent practice. However, the impact of resident involvement on patient outcomes, particularly complications following hip fracture fixation, remains unclear. This study evaluates whether resident participation in intertrochanteric and femoral neck fracture surgical treatments only influences postoperative complication rates.

**Materials and Methods:**

We retrospectively analyzed de-identified data from the American College of Surgeons National Surgical Quality Improvement Program (ACS-NSQIP) from 2005 to 2012. A total of 6300 patients who underwent hip fracture fixation were identified using ICD-9-CM codes, after excluding incomplete cases. Patients were divided into two cohorts: procedures performed by attending surgeons alone (AA) and those with resident involvement (AR). Groups were matched using propensity score analysis. Postoperative complications were compared using multivariate logistic regression, adjusting for potential confounders.

**Results:**

In the AR group, 45.0% of patients experienced at least one complication, compared to 29.3% in the AA group. The AR group had higher rates of postoperative transfusions and systemic complications, including sepsis. Wound dehiscence and organ space infections were significantly more common in the AR group (*P* < 0.02). Additionally, the AR group demonstrated longer operation times, extended anesthesia-to-incision intervals, and prolonged hospital stays. When stratified by fracture type, intertrochanteric fractures were associated with significantly higher complication rates compared to femoral neck fractures in the AR group (*P* < 0.001). Despite these findings, multivariate regression analysis revealed no significant difference in the rate of serious complications; including cardiopulmonary, neurologic, infectious, renal, and mortality events; between groups.

**Conclusion:**

Resident involvement in hip fracture fixation is associated with increased overall complication rates, particularly in intertrochanteric fractures, but does not significantly affect the incidence of serious complications. These findings highlight opportunities to improve resident training, particularly in surgical efficiency, patient positioning, hemostasis, closure technique, and perioperative care, to better prepare residents for independent surgical practice.

## Introduction

The prevalence of hip fractures has been increasing throughout the years in the USA due to the aging population. The annual incidence of hip fractures is estimated to double in the next 25 years [1, 2]. The most common sites affected by hip fractures are the intertrochanteric and femoral neck regions [1].

Surgical residency programs are critical to developing the next generation of surgeons, ensuring they gain the skills, knowledge, and experience required for independent practice. However, there are ongoing concerns about the impact of resident involvement on patient outcomes, particularly for hip fractures surgical procedure. The balance between providing adequate hands-on experience for residents and maintaining high patient safety standards presents a challenging dynamic in surgical education.

Several studies have explored the effect of resident involvement on surgical outcomes, with mixed results [2–5]. Kiran et al. found that resident participation in surgical procedures was associated with a longer operative time and increased complication rates, including wound infections and reoperations [2]. This is consistent with other literature showing that resident involvement increases the complication rate in total hip arthroplasty [5]. Other studies have shown that resident involvement did not correlate with an increased rate of complications following lower extremity total joint arthroplasty or intertrochanteric hip fracture fixation [5–7].

To our knowledge, no studies have evaluated the impact of surgical residents on complication rates in the treatment of both intertrochanteric and femoral neck fractures. While Neuwirth et al. examined resident participation in intertrochanteric fractures, their study was limited by a small sample size [5]. This study aims to compare surgical outcomes between procedures performed solely by attending surgeons and those involving residents, identifying potential gaps in residency training that may contribute to unfavorable events.

## Materials and methods

A retrospective cohort study (Level of Evidence III) was performed using the American College of Surgeons database, the National Surgical Quality Improvement Project (NSQIP). The de-identified data is prospectively collected 30 days post-operation, and patients lost to follow-up before this period are not included in the database [8]. Previous publications on the NSQIP database were referenced for relevant variables and general methodology [9–12]. We used STROBE (Strengthening the Reporting of Observational Studies in Epidemiology) as the assessment tool.

Data from the NSQIP Participant Use Files (2005–2012) were extracted for secondary analysis, as NSQIP discontinued tracking resident participation after 2012. Patients were identified using the following Current Procedural Terminology (CPT) codes: Femoral intertrochanteric fractures (27,244, 27,245), Femoral neck fractures (27,236, 27,235), and hip arthroplasty (27,125, 27,130). Subsequently, the International Classification Diseases, 9th Revision, Clinical Modification (ICD-9-CM) codes were used to identify patients undergoing the procedures exclusively for intertrochanteric fractures (820.21; 820.31) and femoral neck fractures (820.00–820.19; 820.8; 820.9), thereby restricting the cohort to fracture-related cases only. Only cases with complete surgical data on trainee participation were included in the analysis. This study did not require Institutional Review Board approval at our institution, given de-identified data.

Resident involvement in the surgical treatment of femoral neck and intertrochanteric fractures was examined as a binary variable: the attending-alone (AA) group and the Attending-and-Residents (AR) group. Data on relevant demographic characteristics, important risk factors were extracted and shown in detail in Table 1 before matching.

**Table 1:** Demographic characteristics and risk factors of patients in the Attending-Alone and Attending and Residents groups

Characteristics	Attending-Alone (*n* = 5033)	Attending-and-Residents (*n* = 1267)	*P*-value
*Fracture type, n (%)*			0.59
Intertrochanteric	2078 (41.3%)	512 (40.4%)	
Femoral neck	2955 (58.7%)	755 (59.6%)	
Age, years, mean ± SD	81.90 ± 12.67	79.84 ± 14.10	** < 0.001**
*Age group, years, n (%)*			** < 0.001**
< 70	758 (15.1%)	278 (21.9%)	
≥ 70	4275 (84.9%)	989 (78.1%)	
Female	3586 (71.3%)	851 (67.3%)	**0.005**
BMI, mean ± SD	24.80 ± 5.77	24.77 ± 5.61	0.91
*BMI, category, n (%)*			0.3
< 25 (Normal weight)	2940 (58.5%)	746 (59.0%)	
≥ 25–< 30 (Overweight)	1410 (28.10%)	329 (26.0%)	
≥ 30–< 40 (Obese)	595 (11.8%)	170 (13.4%)	
≥ 40 (Morbidly Obese)	77 (1.5%)	19 (1.5%)	
Race	4587	1043	** < 0.001**
Caucasian	4424 (96.4%)	957 (91.8%)	
African American	163 (3.6%)	86 (8.2%)	
*Risk factors*			0.52
ASA classification, n(%)			
1	48 (1.0%)	14 (1.1%)	
2	915 (18.2%)	221 (17.4%)	
3	3141 (62.5%)	779 (61.5%)	
4	917 (18.5%)	252 (19.9%)	
*Anesthesia type, n (%)*			** < 0.001**
General	3748 (74.5%)	1026 (81.0%)	
Spinal	1243 (24.8%)	229 (18.1%)	
*Diabetes, n (%)*			0.6
No DM	4106 (81.6%)	1044 (82.4%)	
Non-insulin-dependent DM	542 (10.8%)	124 (9.8%)	
Insulin-dependent DM	385 (7.6%)	99 (7.8%)	
Alcohol use, n (%)	158 (3.4%)	52 (4.5%)	0.07
Smoking, n (%)	569 (11.3%)	174 (13.7%)	**0.02**
*Functional status, n (%)*			** < 0.001**
Independent	2731 (54.3%)	782 (61.7%)	
Partially dependent	1799 (35.7%)	424 (33.3%)	
Totally dependent	474 (9.4%)	58 (4.6%)	
Hemiplegia, n (%)	123 (2.6%)	31 (2.7%)	1
COPD, n (%)	608 (12.1%)	157 (12.4%)	0.77
MI within 6 months, n (%)	74 (1.6%)	38 (3.3%)	** < 0.001**
CHF, n (%)	171 (3.4%)	55 (4.3%)	0.11
HTN, n (%)	3584 (71.2%)	881 (69.5%)	0.25
Peripheral vascular disease, n (%)	138 (2.9%)	43 (3.7%)	0.19
Cerebrovascular accidents, n (%)	940 (20.0%)	210 (18.1%)	0.15
Liver disease, n (%)	15 (0.3%)	10 (0.9%)	**0.002**
Chronic steroid use, n (%)	236 (4.7%)	76 (6.0%)	0.06
Current cancer, n (%)	97 (2.1%)	33 (2.8%)	0.12
Disseminated cancer, n (%)	69 (1.4%)	25 (2.0%)	0.12
Dialysis, n (%)	93 (1.8%)	52 (4.1%)	** < 0.001**
Bleeding disorders, n (%)	897 (17.8%)	222 (17.5%)	0.81
*Modified Charlson index, n (%)*			** < 0.001**
0	60 (1.3%)	22 (1.9%)	
1	106 (2.3%)	49 (4.2%)	
2	270 (5.7%)	87 (7.5%)	
3	596 (12.7%)	149 (12.8%)	
4	1902 (40.5%)	430 (37.0%)	
5	874 (18.6%)	181 (15.6%)	
≥ 6	893 (19.0%)	244 (21.0%)	
Transfusion > 4 units PRBCs in 72 h before surgery, n (%)	166 (3.3%)	53 (4.2%)	0.14
Anesthesia start to surgery start (mins*), mean ± SD	38.95 ± 15.87	49.08 ± 19.27	** < 0.001**
Operative time (mins), mean ± SD	60.60 ± 33.02	80.28 ± 36.89	** < 0.001**
*Wound classification, n (%)*			**0.03**
clean	4956 (98.5%)	1258 (99.3%)	
others	77 (1.5%)	9 (0.7%)	
Serum albumin g/dl*, mean ± SD (n)	3.51 ± 0.55 (2979)	3.42 ± 0.61 (592)	**0.001**
Serum albumin group, n (%)	2979	592	** < 0.001**
Normal (≥ 3.5–< 5.5)	1715 (57.6%)	288 (48.6%)	
Hypoalbuminemia (< 3.5)	1259 (42.3%)	303 (51.2%)	
Hyperalbuminemia (≥ 5.5)	5 (0.2%)	1 (0.2%)	
Hematocrit, mean ± SD (n)	35.53 ± 5.10	34.99 ± 5.15	**0.001**
*Hematocrit categories, n (%)*			**0.04**
Normal (39–49)	1245 (25.2%)	254 (21.6%)	
Low (< 39)	3681 (74.4%)	914 (77.9%)	
High (> 49)	22 (0.4%)	6 (0.5%)	
Creatinine g/dl, mean ± SD	1.11 ± 0.83	1.16 ± 1.03	0.1
INR**, mean ± SD (n)	1.14 ± 0.31 (4177)	1.11 ± 0.24 (1528)	** < 0.001**
PTT**, mean ± SD (n)	29.36 ± 6.63 (3209)	29.11 ± 6.20 (936)	0.3
Prior Operation within 30 days, n (%)	51 (1.1%)	7 (0.6%)	0.18
Discharge destination other than home, n (%)	1363/1770 (77.0%)	643/847 (75.9%)	0.28
Total length of stay, mean ± SD (n)	6.69 ± 7.43	7.73 ± 9.19	** < 0.001**
*Total length of stay categories, n (%)*			** < 0.001**
≤ 14 days	4768 (94.8%)	1137 (90.3%)	
> 14 days	262 (5.2%)	122 (9.7%)	

*SD: Standard Deviation, mins: minutes, g/dl: gram per deciliters

**BMI: Body Mass Index, ASA: American Scientists of Anesthesiologists physical status, DM: Diabetes Mellitus, COPD: Chronic Obstructive Pulmonary Disease, MI: Myocardial Infarction, CHF: Congestive Heart Failure, HTN: Hypertension, INR: International Normalized Ratio, PTT: Partial Thromboplastin Time. Bold values indicate statistical significance

**Fig. 1:**
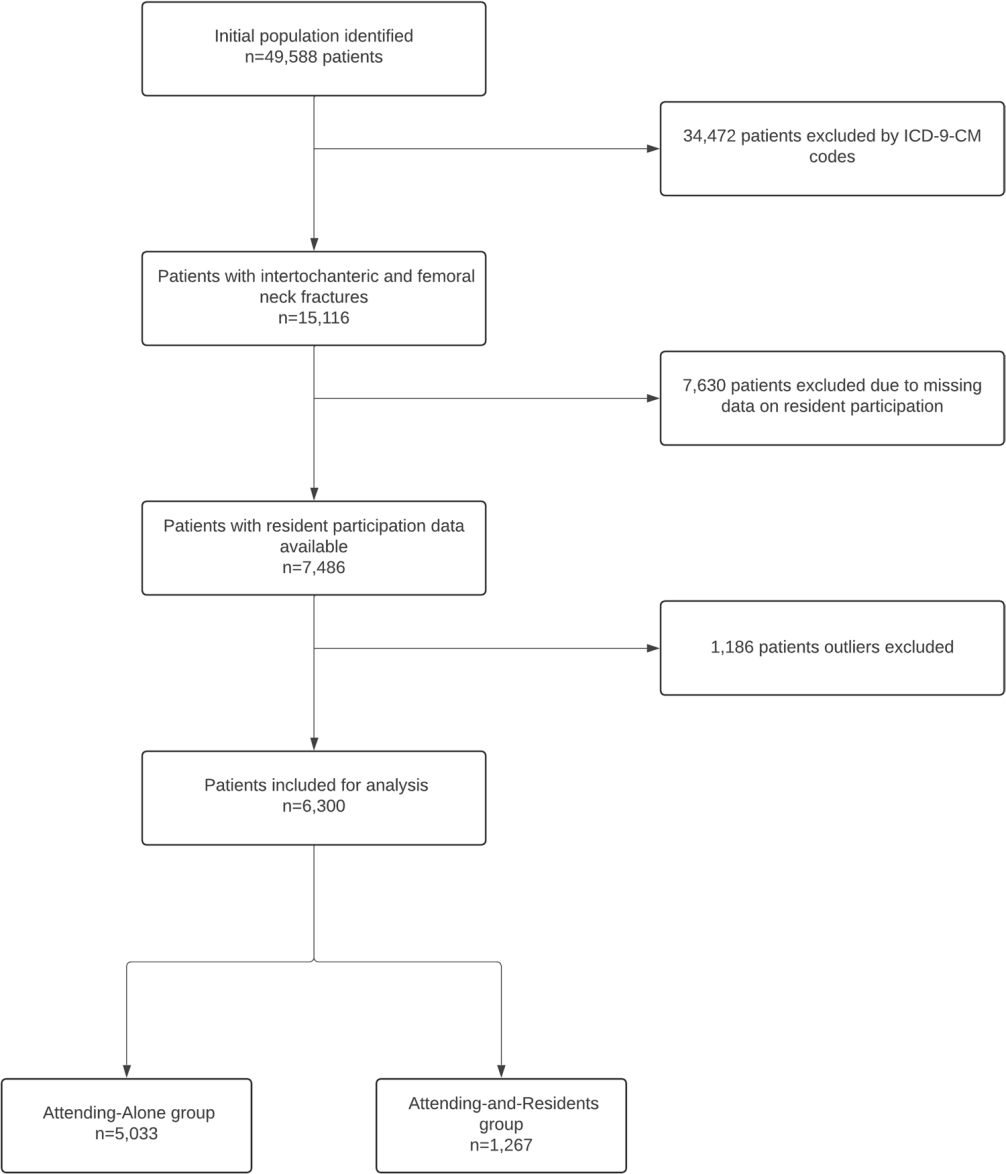
Flow Diagram of Patient Enrollment and Final Study Cohort

Outliers in key demographics and risk factors variables, including BMI, Age, ASA 5, Operation Time, Anesthesia start to surgery start time, Hematocrit level, Albumin level, and Total length of stay, were identified using the SPSS box plot method (values > 1.5 × Interquartile Range) and excluded to reduce skewing of data analysis. Finally, 6300 patients were included in the secondary analysis. The process of patient inclusion and exclusion is shown in Fig. 1.

The modified Charlson Comorbidity Index (mCCI) was computed based on established literature and encompassed 11 variables [6, 7, 9]. Cerebrovascular Disease (1 point) is defined as a history of transient ischemic attack or cerebrovascular accident, with or without neurological deficits. Chronic Pulmonary Disease (1 point), Congestive Heart Failure (1 point), and Myocardial Infarction (1 point). A history of revascularization, angioplasty, or rest pain defines Peripheral Vascular Disease (1 point). Diabetes Mellitus (2 points) covers both insulin-dependent and non-insulin-dependent types. Hemiplegia and Renal Disease (2 points each), with the latter defined as the current use of dialysis. The presence of ascites or esophageal varices characterizes Liver Disease (3 points). Age categories are scored as follows: 0 points for ages < 50, 1 point for ages 50–59, 2 points for ages 60–69, 3 points for ages 70–79, and 4 points for ages ≥ 80. And 6 points for Disseminated Cancer.

The primary outcome was the incidence of any postoperative complications. This was further subdivided into overall systemic, serious systemic, and local complications. Systemic complications involved postoperative unfavorable events and medical complications. In the tables, a dividing line was included to separate serious systemic complications (reported above the line) from overall systemic complications (which include both the serious systemic events and those listed below the line). Whereas local complications involved local surgical complications and re-operation, as detailed in Table 2. Another outcome of interest investigated was extended length of stay, defined as more than 14 days of hospital stay following the surgery. Intraoperative complications, although captured in the NSQIP database, were excluded from this analysis, as the study focused specifically on postoperative complications occurring within 30 days of surgery.

**Table 2:** Comparison of Different Postoperative Complication Types Between the Attending-Alone and Attending-and-Residents Groups

Characteristics of overall systemic complications	Attending-Alone (*n* = 5033)	Attending-and-Residents (*n* = 1267)	*P*-value
Overall systemic complication	1573 (31.3%)	544 (42.9%)	** < 0.001**
Serious systemic complications, n(%)	508 (10.1%)	131 (10.3%)	0.84
Re-intubation, n(%)	72 (1.4%)	24 (1.9%)	0.25
Ventilator > 48 h, n (%)	30 (0.6%)	9 (0.7%)	0.68
MI, n (%)	78 (1.5%)	31 (2.4%)	**0.03**
Cerebrovascular accident, n (%)	24 (0.5%)	11 (0.9%)	0.14
Coma for > 24 h, n (%)	7 (0.1%)	6 (0.5%)	**0.03**
Cardiac arrest requiring CPR, n (%)	44 (0.9%)	9 (0.7%)	0.61
Sepsis, n (%)	79 (1.6%)	29 (2.3%)	0.09
Septic shock, n (%)	45 (0.9%)	6 (0.5%)	0.14
Pulmonary embolism, n (%)	31 (0.6%)	7 (0.6%)	0.84
Renal failure, n (%)	22 (0.4%)	4 (0.2%)	0.64
Death, n (%)	287 (5.7%)	64 (5.1%)	0.37
Post-op transfusion, n (%)	913 (18.1%)	434 (34.3%)	** < 0.001**
Renal insufficiency, n (%)	20 (0.4%)	3 (0.2%)	0.6
Pneumonia, n (%)	166 (3.3%)	42 (3.3%)	1
UTI*, n (%)	294 (5.8%)	69 (5.4%)	0.59
DVT*, n (%)	56 (1.1%)	9 (0.7%)	0.21
Characteristics of local complications	Attending-Alone (*n* = 5033)	Attending-and-Residents (*n* = 1267)	***P*-value**
Any local complication	183 (3.6%)	56 (4.4%)	0.22
SSI*, n (%)	57 (1.1%)	14 (1.1%)	1
DSSI*, n (%)	16 (0.3%)	7 (0.6%)	0.2
Organ space infection, n (%)	10 (0.2%)	8 (0.6%)	**0.02**
Peripheral nerve injury, n (%)	2 (0.1%)	0 (0.0%)	1
Prosthesis failure, n (%)	2 (0.1%)	1 (0.1%)	1
Wound dehiscence, n (%)	2 (0.1%)	4 (0.3%)	**0.02**
Re-operation, n (%)	135 (2.7%)	40 (3.2%)	0.39
	Attending-Alone (*n* = 5033)	Attending-and-Residents (*n* = 1267)	***P*-value**
Any complication, n (%)	1681 (33.4%)	570 (45.0%)	** < 0.001**

^*^UTI: Urinary Tract Infection, DVT: Deep Vein Thrombosis, SSI: Superficial Surgical Site Infection, DSSI: Deep Surgical Site Infection. Bold values indicate statistical significance

### Statistical methods

#### Statistical analysis was conducted using SPSS version 29.02.

Propensity score matching was applied to minimize confounding bias between the AA and AR cohorts. Cases were matched 1:1 using the nearest neighbor matching algorithm without replacement, with a match tolerance of 0.1. Matching was based on the mCCI. The normality of variables was assessed using skewness and kurtosis tests. Summary statistics were calculated to describe the variables, including means, standard deviations, and percentages. Mean differences in continuous variables were evaluated using the independent t-test, while associations between groups for categorical variables were analyzed using the chi-squared test for both unmatched and matched cohorts.

Binary logistic regression was performed to assess the unadjusted odds ratios between groups for the incidence of complications and extended length of stay. Multivariate logistic regression was used to fit two models: the first model was adjusted for significant demographic characteristics, while the second model further adjusted for important risk factors and fracture types. Variables not included in the first and second models were deemed not clinically or statistically significant.

Fracture type was investigated as a potential confounder in the incidence of complications between groups by comparing the odds ratios of the general sample population to those of the stratified sample based on fracture types.

Statistical significance for all analyses was set at a *p*-value of less than 0.05.

## Results

Using the NSQIP database, we initially identified 15,116 patients who had undergone surgery for intertrochanteric or femoral neck hip fractures between 2005 and 2012. Data on resident involvement was available for 7486 patients (49.5%). After applying additional exclusion criteria, 6300 patients were included in the analysis: 5033 in the AA group and 1267 in the AR group (Fig. 1).

In both groups, more than half of the patients were female (71.3% in the AA group and 67.3% in the AR group). Most hip fractures were femoral neck fractures (58.7% in the AA group and 59.6% in the AR group; *P*-value = 0.59). The majority of patients were over 70 years old and Caucasian. More than 75% of patients were discharged home in both groups. Significant differences were observed between the groups in operative time (60.60 min for AA vs. 80.28 min for AR; *P*-value < 0.001), time from anesthesia start to surgery start (38.95 min for AA vs. 49.08 min for AR; *P*-value < 0.001), and total length of stay (6.69 days for AA vs. 7.73 days for AR; *P*-value < 0.001). Moreover, a significant increase in operative time was seen whenever residents were present during the operation (60.6 vs 80.28; *P*-value < 0.001). A detailed overview of baseline characteristics and risk factors is described in Table 1.

Regarding postoperative complications, the analysis showed a complication rate of 45.0% in the AR group compared to 33.4% in the AA group (*P*-value < 0.001). Systemic complications were notably higher in the AR group (42.9%) than in the AA group (31.3%). Significant differences were observed in the incidence of postoperative myocardial infarction (MI) (2.5% vs. 1.4%, *P*-value = 0.03), coma lasting more than 24 h (0.5% vs. 0.1%, p = 0.03), and postoperative blood transfusion (34.3% vs. 18.1%, *p* < 0.001).

However, serious systemic complications did not differ significantly between the groups. Local complications showed no significant differences; however, organ space infections and wound dehiscence were significantly more common in the AR group compared to the AA group, as detailed in Table 2.

### Propensity score matched cohort analysis

Propensity score matching produced a cohort of 2329 patients, with 1162 cases in the AA group and 1267 in the AR group. Table 3 indicates that after matching, significant differences persisted for gender, race, anesthesia type, functional health status, myocardial infarction, and dialysis. Additionally, a significant difference emerged post-matching regarding fracture type, which was not observed prior to matching. Significant differences in anesthesia start-to-surgery start time (39.93 min vs. 49.08 min) and operative time (63.24 min vs. 80.28 min) between the AA and AR groups were still present. Patients in the AR group had higher total length of stay (TLOS) compared to the patients in the AA group. Additionally, in the subgroup of patients who received postoperative blood transfusions, those in the resident cohort had a significantly longer total length of stay compared to the attending-only cohort (8.49 ± 11.73 vs. 7.00 ± 5.66 days, *P* = 0.04).

**Table 3:** Demographic characteristics and risk factors of patients in the Attending-Alone and Attending and Residents groups after propensity score matching

Characteristics	Attending Alone (*n* = 1162)	Attending and Residents (*n* = 1267)	*P*-value
*Fracture type, n (%)*			** < 0.001**
Intertrochanteric	353 (30.4%)	512 (40.4%)	
Femoral neck	809 (69.6%)	755 (59.6%)	
Inpatient, n (%)	1150 (99.0%)	1260 (99.4%)	0.25
Age, years, mean ± SD	80.43 ± 13.58	79.84 ± 14.10	0.30
*Age group, years, n (%)*			0.25
< 70	232 (20.0%)	278 (21.9%)	
≥ 70	930 (80.0%)	989 (78.1%)	
Female	841 (72.5%)	851 (67.3%)	**0.005**
BMI, mean ± SD	24.47 ± 5.31	24.77 ± 5.61	0.17
*BMI, category, n (%)*			0.42
< 25 (Normal weight)	695 (59.9%)	746 (59.0%)	
≥ 25–< 30 (Overweight)	318 (27.4%)	329 (26.0%)	
≥ 30–< 40 (Obese)	133 (11.5%)	170 (13.4%)	
≥ 40 (Morbidly Obese)	14 (1.2%)	19 (1.5%)	
Race	4587	1043	** < 0.001**
Caucasian	1015 (96.1%)	957 (91.8%)	
African American	41 (3.9%)	86 (8.2%)	
*Risk factors*			
ASA classification, n (%)			0.59
1	18 (1.6%)	14 (1.1%)	
2	219 (18.9%)	221 (17.4%)	
3	705 (60.8%)	779 (61.5%)	
4	218 (18.8%)	252 (19.9%)	
*Anesthesia type, n (%)*			**0.003**
General	911 (78.4%)	1054 (83.2%)	
Spinal	251 (21.6%)	213 (16.8%)	
*Diabetes, n (%)*			0.38
No DM	932 (80.2%)	1044 (82.4%)	
Non-insulin dependent DM	129 (11.1%)	124 (9.8%)	
Insulin dependent DM	101 (8.7%)	99 (7.8%)	
Alcohol use, n (%)	40 (3.4%)	52 (4.5%)	0.24
Smoking, n (%)	158 (13.6%)	174 (13.7%)	0.95
*Functional status, n (%)*			** < 0.001**
Independent	604 (52.4%)	782 (61.9%)	
Partially dependent	429 (37.2%)	424 (33.5%)	
Totally dependent	120 (10.4%)	58 (4.6%)	
Hemiplegia, n (%)	41 (3.5%)	31 (2.7%)	0.28
COPD, n (%)	139 (12.0%)	157 (12.4%)	0.76
MI within 6 months, n (%)	14 (1.2%)	38 (3.3%)	** < 0.001**
CHF, n (%)	40 (3.4%)	55 (4.3%)	0.30
HTN, n (%)	776 (66.8%)	881 (69.5%)	0.15
Peripheral vascular disease, n (%)	32 (2.8%)	43 (3.7%)	0.24
Cerebrovascular accidents, n (%)	228 (19.6%)	210 (18.1%)	0.37
Liver disease, n (%)	3 (0.3%)	10 (0.9%)	0.09
Chronic steroid use, n (%)	50 (4.3%)	76 (6.0%)	0.07
Current cancer, n (%)	22 (1.9%)	33 (2.8%)	0.17
Disseminated cancer, n (%)	17 (1.5%)	25 (2.0%)	0.35
Dialysis, n (%)	24 (2.1%)	52 (4.1%)	**0.005**
Bleeding disorders, n (%)	184 (15.8%)	222 (17.5%)	0.28
*Modified Charlson index, n (%)*			1.0
0	22 (1.9%)	22 (1.9%)	
1	49 (4.2%)	49 (4.2%)	
2	87 (7.5%)	87 (7.5%)	
3	149 (12.8%)	149 (12.8%)	
4	430 (37.0%)	430 (37.0%)	
5	181 (15.6%)	181 (15.6%)	
≥ 6	244 (21.0%)	244 (21.0%)	
Transfusion > 4 units PRBCs in 72 h before surgery, n (%)	28 (2.4%)	53 (4.2%)	**0.02**
Anesthesia start to Surgery start (mins), mean ± SD	38.93 ± 16.67	49.08 ± 19.27	** < 0.001**
Operative time (mins), mean ± SD	63.24 ± 33.67	80.28 ± 36.89	** < 0.001**
*Wound classification, n (%)*			0.51
Clean	1150 (99.0%)	1258 (99.3%)	
Others	12 (1.0%)	9 (0.7%)	
Preoperative Serum albumin g/dl, mean ± SD (n)	3.50 ± 0.54 (654)	3.42 ± 0.61 (592)	**0.02**
Preoperative Serum albumin group, n (%)	2979	592	**0.03**
Normal (≥ 3.5–< 5.5)	363 (55.5%)	288 (48.6%)	
Hypoalbuminemia (< 3.5)	290 (44.3%)	303 (51.2%)	
Hyperalbuminemia (≥ 5.5)	1 (0.2%)	1 (0.2%)	
Preoperative Hematocrit, mean ± SD (n)	35.96 ± 4.95	34.99 ± 5.15	** < 0.001**
*Preoperative Hematocrit categories, n (%)*			**0.002**
Normal (39–49)	315 (27.9%)	254 (21.6%)	
Low (< 39)	811 (71.7%)	914 (77.9%)	
High (> 49)	5 (0.4%)	6 (0.5%)	
Preoperative Creatinine g/dl, mean ± SD	1.13 ± 0.97	1.16 ± 1.03	0.41
Preoperative INR, mean ± SD (n)	1.12 ± 0.27 (962)	1.11 ± 0.24 (1165)	0.14
Preoperative PTT, mean ± SD (n)	29.77 ± 6.97 (724)	29.11 ± 6.20 (936)	**0.04**
Prior Operation within 30 days, n (%)	51 (1.1%)	7 (0.6%)	0.18
Discharge Destination Other than home, n (%)	265/371 (71.4%)	643/847 (75.9%)	0.10
Total Length of Stay, mean ± SD (n)	6.58 ± 6.72	7.73 ± 9.19	** < 0.001**
*Total length of stay categories, n (%)*			** < 0.001**
≤ 14 days	1101 (94.8%)	1137 (90.3%)	
> 14 days	60 (5.2%)	122 (9.7%)	

Bold values indicate statistical significance

Regarding complications, significant differences were observed in overall and systemic complications, specifically cerebrovascular accidents, sepsis, and postoperative blood transfusion. Although the proportions of organ-space infections and wound dehiscence were similar before matching, statistical significance was not achieved post-matching, likely due to the reduced power resulting from the smaller sample size after matching (Table 4).

**Table 4:** Comparison of Different Postoperative Complication Types Between the Attending-Alone and Attending-and-Residents Groups after propensity score matching

Characteristics of overall systemic complications	Attending Alone (*n* = 1162)	Attending and Residents (*n* = 1267)	Effect size	*P*-value
Overall systemic complication	321 (27.6%)	544 (42.9%)	0.20	** < 0.001**
Serious systemic complications, n (%)	106 (9.1%)	131 (10.3%)	0.2	0.34
Re-intubation, n (%)	17 (1.5%)	24 (1.9%)		0.44
Ventilator > 48 h, n (%)	6 (0.5%)	9 (0.7%)		0.61
MI, n (%)	18 (1.5%)	31 (2.4%)		0.15
Cerebrovascular accident, n (%)	3 (0.3%)	11 (0.9%)	0.04	**0.047**
Coma for > 24 h, n (%)	1 (0.1%)	6 (0.5%)		0.13
Cardiac arrest requiring CPR, n (%)	8 (0.7%)	9 (0.7%)		1.0
Sepsis, n (%)	14 (1.2%)	29 (2.3%)	0.04	**0.046**
Septic shock, n (%)	10 (0.9%)	6 (0.5%)		0.32
Pulmonary embolism, n (%)	7 (0.6%)	7 (0.6%)		1.0
Renal Failure, n (%)	6 (0.5%)	4 (0.3%)		0.53
Death, n (%)	55 (4.7%)	64 (5.1%)		0.78
Post-op transfusion, n (%)	167 (14.4%)	434 (34.3%)	0.23	** < 0.001**
Renal insufficiency, n (%)	5 (0.4%)	3 (0.2%)		0.49
Pneumonia, n (%)	30 (2.6%)	42 (3.3%)		0.34
UTI, n (%)	64 (5.5%)	69 (5.4%)		1.0
DVT, n (%)	10 (0.9%)	9 (0.7%)		0.82
Characteristics of local complications	Attending alone (*n* = 1162)	Attending and residents (*n* = 1267)	Effect size	*P*-value
Local complication	36 (3.1%)	56 (4.4%)	0.1	0.09
SSI, n (%)	11 (0.9%)	14 (1.1%)		0.84
DSSI, n (%)	4 (0.3%)	7 (0.6%)		0.55
Organ space infection, n (%)	2 (0.2%)	8 (0.6%)		0.11
Peripheral nerve injury, n (%)	1 (0.1%)	0 (0.0%)		0.48
Prosthesis failure, n (%)	2 (0.1%)	1 (0.1%)		1
Wound dehiscence, n (%)	1 (0.1%)	4 (0.3%)		0.38
Re-operation, n (%)	25 (2.2%)	40 (3.2%)		0.13
	Attending Alone (*n* = 1162)	Attending and Residents (*n* = 1267)	Effect size	*P*-value
Any complications, n (%)	341 (29.3%)	570 (45.0%)	0.20	** < 0.001**

Bold values indicate statistical significance

**Fig. 2:**
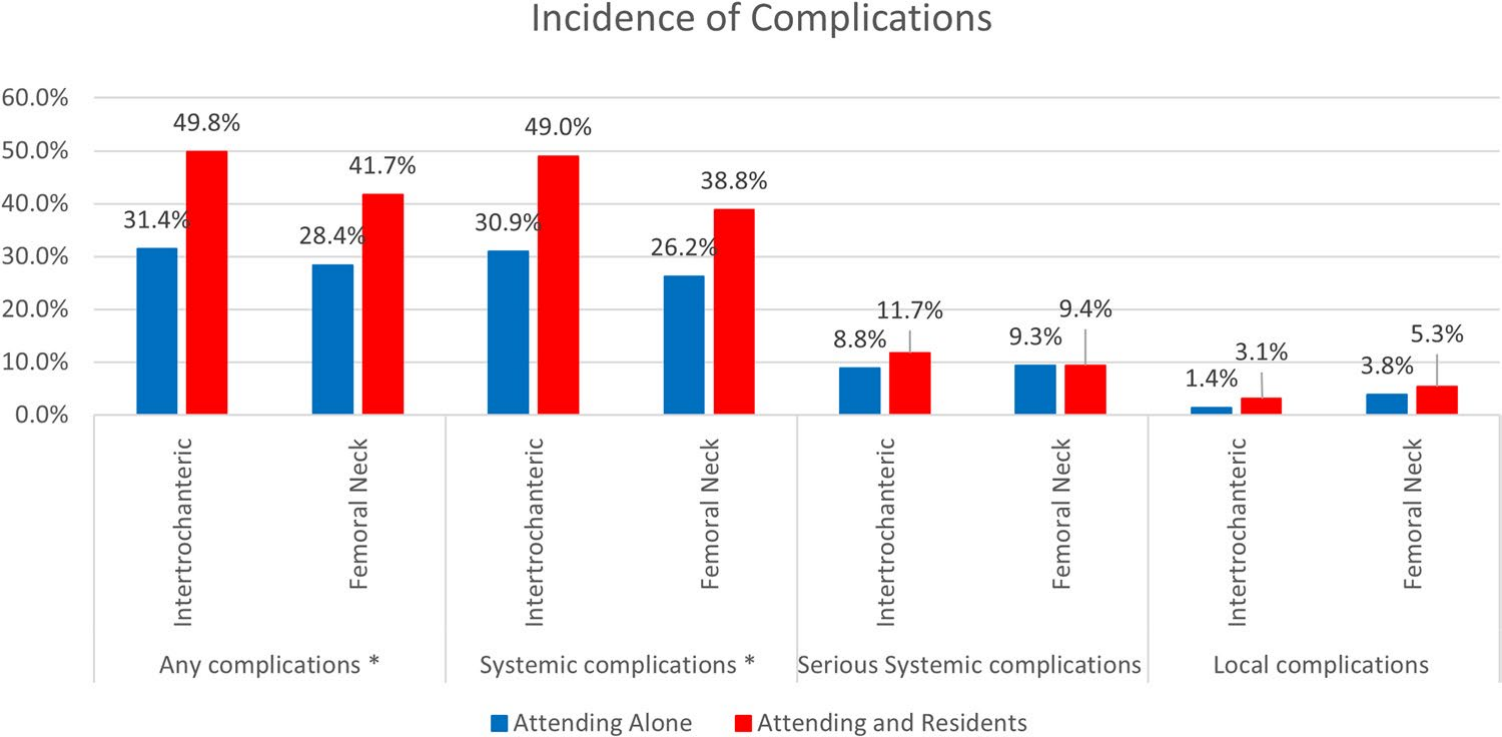
Incidence of complications by hip fracture type between the groups (*: statistical significance observed between the groups)

Figure 2 illustrates that the incidence of any complications and systemic complications was significantly higher in the AR group compared to the AA group for both types of hip fractures. However, serious systemic and local complications did not differ significantly between the groups for either fracture type. Furthermore, intertrochanteric fractures were associated with a higher overall incidence of complications compared to femoral neck fractures in all types of complications.

Logistic regression interaction testing, using the product of the variables of interest, showed that fracture type did not confound the relationship between resident involvement and complication rates. As shown in Table 5, fracture type had no significant impact on this relationship. The overall unadjusted OR was 1.97, with an OR of 2.16 for intertrochanteric fractures and 1.80 for femoral neck fractures, all with overlapping confidence intervals.

**Table 5:** Odds of any complications in hip fracture surgery with resident involvement vs. no involvement stratified by fracture type

Variables	Resident involvement Yes vs No	95% CI	*P*-value
OR any complications overall	1.969	1.665–2.329	< 0.001
OR any complications intertrochanteric fractures	2.163	1.629–2.873	< 0.001
OR any complications femoral neck fractures	1.802	1.460–2.224	< 0.001

^*^CI: Confidence Interval, OR: Odds Ratio

After adjusting for relevant demographics and risk factors, two additional models were developed using the variables outlined in Table 6. Across all logistic regression models, the OR for the incidence of any complications remained approximately 1.82, indicating 82% higher odds of complications with resident involvement. However, no significant increase was observed in the incidence of serious systemic complications. All models consistently showed a significant increase in the likelihood of an extended total length of stay (greater than 14 days) and prolonged operative time (≥ 90 min), with ORs of approximately 2.0 and 2.38, respectively, when residents were involved (Table 6). Additional analysis using the adjusted Model 2 regression, patients with extended operative time had 1.29 times higher odds of developing any complication compared to those without extended time (OR 1.29, 95% CI: 1.05–1.58, *P* = 0.02).

**Table 6:** Bivariate and Multivariate Logistic Regression Models Comparing AR to AA Group with the Incidence of Complications and Extended Length of Stay

	Unadjusted OR (95% CI)	*P*-value	Adjusted 1 OR (95% CI)	*P*-value	Adjusted 2 OR (95% CI)	*P*-value
Resident involvement Yes vs No						
Any complication	1.63 (1.44–1.85)	** < 0.001**	2.01 (1.67–2.41)	** < 0.001**	1.84 (1.42–2.38)	** < 0.001**
Serious systemic complication	1.03 (0.84–1.26)	0.8	1.12 (0.84–1.49)	0.42	1.45 (0.99–2.12)	0.06
Total length of stay > 14 vs ≤ 14	1.95 (1.56–2.44)	** < 0.001**	1.70 (1.13–2.55)	**0.011**	2.48 (1.46–4.23)	** < 0.001**
Operative time ≥ 90 vs < 90	2.49 (2.06–3.00)	** < 0.001**	2.38 (1.94–2.90)	** < 0.001**	2.27 (1.70–3.04)	** < 0.001**

^*^OR: Odds Ratio, CI: Confidence Interval

^**^Adjusted 1: Race, Gender, Age (categorical). Adjusted 2: Adjusted 1 + albumin levels (categorical), HCT levels (categorical), Alcohol history, Smoking Status, Fracture Type. Bold values indicate statistical significance

## Discussion

This study provides valuable insight into the impact of resident involvement on surgical outcomes in the context of hip fracture surgery. Consistent with previous research, our findings indicate that the presence of residents significantly increases operative time and anesthesia duration. The mean operative time in the AR group was 80.28 min compared to 63.24 min in the AA group (*P*-value < 0.001), and the time from anesthesia start to surgery start was also significantly longer in the AR group (49.08 min vs. 38.93 min; *P*-value < 0.001). This aligns with earlier studies, such as the work by Davis et al., which demonstrated that resident involvement adds approximately 20–47% to the operative time in various surgical procedures [8].

The increase in operative time is likely multifactorial. By nature of their training, residents require more time to perform surgical tasks, often performed under the close supervision of attending surgeons. While this extended time allows residents to gain valuable hands-on experience, it also increases patients’ exposure to anesthesia, which has been linked to higher rates of postoperative complications, including cognitive decline and respiratory complications [10, 11]. Despite these concerns, it is important to highlight that serious systemic complications did not significantly differ between our study's AR and AA groups (10.3% vs. 9.1%, *P*-value = 0.34). This suggests that when properly supervised, resident involvement does not elevate the risk of life-threatening complications. Supporting this, other studies investigating resident participation in orthopedic surgery have shown that while the presence of residents may increase operative time, this does not correlate with higher morbidity or mortality rates [5, 13, 14].

The prolonged duration from anesthesia start time to incision time in the AR group is likely multifactorial. Many surgeries involving residents are performed at academic centers, where also anesthesia teams often include trainees still learning their profession. If this delay is indeed multifactorial, it underscores the importance of enhanced training for orthopedic residents in efficient patient positioning, prepping, and draping, as well as improved training for anesthesia residents in central line access, epidural and spinal techniques, and intubation. These measures could help reduce overall anesthesia time.

A notable finding in our study was the higher rate of wound dehiscence in the AR group (0.3% vs. 0.1% in the AA group, *P*-value = 0.02 before matching). Although the proportions remained the same after matching, statistical significance was lost. This complication is often linked to inadequate soft tissue handling and poor wound closure techniques [15]. Implementing focused training programs, such as simulation-based learning and supervised practice in wound closure, could help mitigate these complications and enhance patient outcomes.

Another key finding from our study was the extended TLOS associated with resident involvement. The mean TLOS in the AR group was 7.73 days compared to 6.58 days in the AA group (*P*-value < 0.001). A similar pattern was observed in the subgroup of patients who received postoperative blood transfusions, where resident involvement was also associated with longer TLOS. This finding aligns with previous literature indicating that resident involvement is often associated with more extended hospital stays. While the exact reasons for the extended TLOS are multifactorial, several hypotheses can be proposed. First, residents may adopt a more cautious approach to postoperative management, leading to extended observation periods before discharge. Second, while NSQIP does not capture data on postoperative resident involvement, it is possible that differences in experience with managing postoperative issues such as nutritional deficits, wound care, or rehabilitation may contribute to prolonged recovery. A study by Itani et al. similarly found that resident involvement was linked to more extended hospital stays, particularly in complex cases where postoperative care significantly influenced patient outcomes [16]. Third, academic institutions, where residents actively participate in patient care, may inherently demonstrate lower efficiency in discharging patients than private institutions. This disparity can be attributed to the complexity of these institutions' cases, including patients with higher comorbidities and limited access to resources, both of which can prolong the TLOS. For instance, Siciliani et al. found that patients undergoing elective hip replacement experienced 18% and 40% shorter lengths of stay in private institutions than in National Health Service public hospitals [17]. Similarly, Skura et al. reported that Medicaid patients undergoing lower extremity total joint arthroplasty had significantly longer lengths of stay than those with Medicare or private insurance [18]. Our findings indicate that while residents may increase financial costs to the healthcare system, this does not come at the expense of short-term patient outcomes. To address these challenges, residency programs should prioritize education on efficient patient disposition and consider integrating nutrition courses to prevent postoperative malnutrition, especially in dependent patients.

Moreover, the increased need for postoperative blood transfusions in the AR group (34.3% vs. 14.4% in the AA group, *P*-value < 0.001) likely contributed to the extended TLOS. Blood transfusions, while necessary in some instances, are associated with increased morbidity, including infections, prolonged recovery, and extended hospital stays [19–21]. The higher rate of transfusions in the AR group indicates a need for additional training for residents on techniques to minimize blood loss by improving skills in soft tissue handling and achieving better hemostasis during surgery, residents could potentially reduce the need for transfusions and shorten recovery times.

This study analysis revealed that any complication rates were higher in the AR group (45.0% vs. 29.3%, *P*-value < 0.001); however, there was no significant difference in serious systemic complications. This aligns with prior studies, such as those by Neuwirth et al., which found that resident involvement was associated with increased rates of minor complications, longer operation times, and extended lengths of stay. However, there was no significant difference in 30-day mortality or severe morbidity between the two groups. These findings suggest that while residents may contribute to longer surgeries and a higher incidence of minor complications, their involvement does not compromise overall patient safety when adequate supervision is provided [5].

Another key point to discuss is the ethical duty to balance surgical training with patient safety is grounded in the dual obligations to provide optimal care to individual patients while ensuring the development of competent future surgeons. Structured mentorship, graded responsibility, and appropriate supervision are essential to maintain patient safety while fostering skill acquisition [22, 23].

The limitations of this study include the limited data on resident participation during the analyzed years. Patients in the AR group were more likely to be treated at academic centers, which often differ from non-academic centers in case complexity, the presence of less experienced anesthesia trainees, and a higher prevalence of comorbidities. While matching the cohort using the mCCI aimed to control for these differences, residual confounding may remain. Additionally, the 30-day follow-up period dictated by the NSQIP database restricts the evaluation of long-term outcomes. The absence of variables detailing the extent of resident involvement or their level of training further limits the analysis. Importantly, time from injury to surgery, which can significantly influence length of stay and postoperative outcomes, is not recorded in the NSQIP database and could not be accounted for in this study. Additionally, there is a potential for selection bias, as residents are more likely to be involved in more complex cases even within the same fracture type, while simpler cases are more often managed solely or in private centers by the attending surgeon. This could partially account for the observed differences in outcomes between the two groups. Lastly, the NSQIP database does not distinguish between hospitals or institutions, hindering the ability to perform comparative analyses across centers.

This study highlights several key areas that require enhancement in U.S. residency training programs. It notes increased operative times and a prolonged interval from the start of anesthesia to the commencement of surgery. Additionally, there are higher rates of wound dehiscence, increased postoperative blood transfusion needs, and longer hospital stays associated with resident participation. These findings underscore the urgent need for focused education in perioperative care, surgical techniques, and postoperative management. By addressing these identified gaps, residency programs can better prepare residents with the essential skills to perform surgeries efficiently and safely, thereby improving the overall quality of patient care.

## Data Availability

The anonymized data collected are available via the NSQIP data repository: https://www.facs.org/quality-programs/data-and-registries/acs-nsqip/participant-use-data-file/
